# HSP105 inhibition downregulates store-operated calcium entry and promotes acute UVB-induced tight junction disruption

**DOI:** 10.1371/journal.pone.0314816

**Published:** 2024-12-05

**Authors:** Kaiyi Zhou, Siyu Luo, Qinxiao Wang, Qian Ye, Sheng Fang

**Affiliations:** 1 Department of Dermatology, The First Affiliated Hospital of Chongqing Medical University, Chongqing, China; 2 Department of Dermatology, Plastic Surgery Hospital, Chinese Academy of Medical Sciences, Peking Union Medical College, Beijing, China; Stanford University, UNITED STATES OF AMERICA

## Abstract

**Background:**

Tight junction abnormalities are a common feature of inflammatory skin diseases such as psoriasis and atopic dermatitis and contribute to systemic immune responses. Evidence provided to date suggests that Heat shock protein 105 kDa (HSP105) exhibits significant protective effects in response to destructive external stimuli. However, its role in UV-induced skin tight junction remains to be fully understood.

**Objective:**

To investigate the role and underlying mechanisms of HSP105 in acute UVB-induced tight junction damage.

**Methods:**

By utilizing bioinformatics analysis, together with an in vitro UVB-induced tight junction injury model in HaCaT cells, we investigated the expression and localization of HSP105 and the tight junction proteins CLDN1, CLDN4, and OCLN. The role of HSP105 was further explored through shRNA-mediated silencing and lentiviral overexpression in HaCaT cells. Potential pathways by which HSP105 regulates tight junction were analyzed using the GSEA algorithm and validated through in vitro experiments.

**Results:**

Acute UVB irradiation mainly disrupted the distribution of CLDN1, CLDN4, and OCLN in HaCaT cells, while gene expression remained largely unaffected. Acute UVB irradiation also caused a reduction in HSP105 protein levels in HaCaT cells. Inhibition of HSP105 expression worsened tight junction fragmentation. GSEA analysis showed that Store-operated calcium entry (SOCE) was significantly correlated with HSP105 expression. Silencing HSP105 downregulated STIM1 transcription and inhibited SOCE, leading to further fragmentation of tight junction proteins. Overexpression of HSP105 partially mitigated the damage to tight junction integrity caused by UVB and SOCE inhibition.

**Conclusion:**

HSP105 protects against acute UVB-induced tight junction damage through the regulation of SOCE. Our findings offer new insights into the treatment of skin barrier injury.

## 1. Introduction

Tight junction between epidermal cells form a continuous network of parallel, interconnected membrane chains essential for maintaining skin barrier integrity **[1]**. Abnormal expression of tight junction proteins plays a key role in various diseases involving skin barrier disruption, such as atopic dermatitis and psoriasis **[2,3]**. Damage to tight junction leads to barrier impairment, which is considered a persistent trigger that facilitates the penetration of external antigens and their interaction with local immune cells, potentially leading to systemic immune responses **[4]**.

Tight junction proteins include claudins, occludin, junctional adhesion molecules, and zonula occludens (ZO) proteins. Claudins interact with scaffolding proteins like ZO-1 and ZO-2, which link claudins to actin filaments **[5]**. Occludin is believed to play a regulatory role rather than directly contributing to the formation of tight junction strands **[6]**. Occludin, claudin-1, claudin-4, and ZO-1 are highly expressed in human keratinocytes and contribute to the formation of a functional barrier under high-calcium conditions **[7,8]**. Therefore, tight junctions involving claudins and occludin serve as a valuable model for studying the effects of external stimuli on skin barrier damage.

UV radiation is known to disrupt the distribution of key tight junction proteins, including Claudin-1, Claudin-4, and Occludin, thereby altering epidermal permeability **[8]**. Among the various UV wavelengths, UVB (290–320 nm) has relatively low penetration but is highly cytotoxic, making it a major factor in epidermal damage. UVB induces DNA damage by generating photoproducts such as cyclobutane pyrimidine dimers, 6–4 photoproducts, and their Dewar valence isomers, while also promoting the production of reactive oxygen species in the epidermis, leading to the accumulation of oxidative products like 8-oxo-guanine **[9]**. However, there is currently little research regarding the mechanisms of UVB-induced tight junction damage, and this issue requires immediate attention.

Heat shock proteins can inhibit the activation of UV-induced inflammatory signaling pathways, reduce oxidative stress and DNA damage, thereby mitigating UV damage **[10,11]**. The HSPH/Hsp110 molecular chaperone family, which is crucial in response to external stress, comprises four human members: HSP105, APG2, APG1, and GRP170 **[12–14]**. This protein family participates in nucleotide exchange within the Hsp70-Hsp40 system, alleviates protein folding stress, and plays a critical role in maintaining protein homeostasis **[15]**. Early studies have explored the role of the HSPH/Hsp110 molecular chaperone family in epithelial tight junction proteins. For instance, in MDCK renal epithelial cells, APG2 has been identified as a chaperone protein interacting with ZO-1 and regulates tight junction assembly and epithelial morphogenesis by modulating the folding and function of ZO-1 **[16]**.

Store-operated calcium entry (SOCE) is the primary pathway for Ca2+ to enter non-excitable cells. STIM1 is an endoplasmic reticulum Ca2+ sensor and responds by coupling with the Ca2+ channel Orai1 at the plasma membrane, which opens the channel and initiates cellular Ca2+ influx **[17]**. Under physiological conditions, elevated intracellular Ca2+ stimulates various calcium-dependent signaling cascades and is crucial for cellular functions such as secretion, migration, and gene expression **[18]**. In keratinocytes, calcium functions as an intracellular second messenger and plays a crucial role in cell proliferation and differentiation **[19,20]**. HSP70 has been shown to regulate intracellular calcium overload in cardiomyocytes by inhibiting STIM1, thereby reducing apoptosis **[21]**. Additionally, HSP27 is a chaperone that stabilizes SOCE-related STIM1 **[22]**.

Based on these findings, we hypothesize that HSP105 may modulate UVB-induced tight junction damage. We used high-Ca2+ medium to enhance tight junction formation in HaCaT cells and exposed them to UVB irradiation to simulate skin tight junction damage. By performing bioinformatics analysis and employing shRNA-mediated gene silencing and lentivirus-mediated overexpression techniques, we identified the protective role of HSP105 in UVB-induced tight junction damage. Furthermore, we demonstrated that SOCE is a key pathway by which HSP105 protects the skin barrier. Our results lay the foundation for developing HSP105-based therapies to treat skin barrier injury.

## 2. Materials and methods

### 2.1. Single-cell RNA-sequencing data and microarray data analysis

First, we obtained bulk sequencing data of human skin from acute UVB exposure from the GEO database (GSE41078). This dataset includes 10 UVB-exposed human skin samples and 10 control samples. Biopsy samples (6 mm) of irradiated skin were collected 24 hours after exposure to twice the minimum erythema dose for sequencing [23]. Background correction and normalization of all datasets were performed using the R package “limma” (version 3.54.0). For genes with multiple probes in ID conversion, the average value was used.

We also obtained single-cell RNA sequencing data (GSE173385) from the skin of a male C57BL/6 mouse exposed to chronic UVB irradiation. This mouse was exposed to UVB radiation three times a week, with each exposure delivering approximately 300 mJ/cm^2^, for a total duration of three weeks. Non-irradiated mice served as control. Quality control was performed on individual cells with the following criteria: 1) more than 200 genes expressed per cell, with expression in more than 10 cells; 2) cells expressed between 500 to 5000 genes; 3) mitochondrial gene content was less than 10%; and 4) cells contained more than 1000 unique molecular identifiers (UMIs). Subsequent data processing, including normalization, scaling, and clustering, was conducted using the Seurat package (version 4.4.1). Single cells were then clustered into subpopulations using the ‘FindClusters’ and ‘FindNeighbors’ functions with dim = 20 and resolution = 1. Finally, single cells were visualized using t-SNE (t-distributed Stochastic Neighbor Embedding) for dimensionality reduction. Cells were manually annotated based on previous research [24,25].

The Monocle package (version 2.32.0) was used to analyze pseudotemporal trajectories of the identified keratinocyte subpopulations. The “DDRTree” function with a maximum fraction setting of 2 was used for dimensionality reduction of the cell data. Cell branching and marker gene expression trends were visualized using the “plot_cell_trajectory” and “plot_pseudotime_heatmap” functions.

Gene Set Enrichment Analysis (GSEA) allows the assessment of the enrichment strength of pathways by analyzing the distribution of a predefined set of genes across a ranked gene list. In this study, we used GSEA to assess the signaling pathways associated with *HSP105*. Keratinocytes were categorized into high and low *HSP105* expression groups based on the median expression level of *HSP105*. The differential gene expression between the two groups was calculated, and all genes were subsequently ranked according to their log2FC values. MSigDB (https://www.gsea-msigdb.org/gsea/msigdb/index.jsp) was selected as the reference gene set [26].

### 2.2. Cell culture

HaCaT cells were purchased from the Chinese Academy of Sciences (Shanghai, China) and cultured in EpiLife cell culture medium (Thermo Fisher Scientific) containing 0.06 mM Ca^2+^, supplemented with 10% fetal bovine serum, 1% penicillin, and streptomycin (Gibco, Carlsbad, USA). Cells were cultured under standard conditions in a humidified incubator with 5% CO_2_ at 37°C. When cells reached approximately 80% confluence, they were transferred to a medium with 1.8 mM Ca^2+^ and incubated for an additional 96 hours to induce intercellular junction formation.

### 2.3. Cell viability calculation

Cell viability following UVB irradiation was assessed using the CCK-8 kit (Beyotime, Jiangsu, China) in accordance with the manufacturer’s instructions. A total of 100 μl containing approximately 5000 cells was seeded into each well of a 96-well plate. Following incubation, 10 μl of CCK-8 solution was added to each well, and the plate was incubated for 1 hour at 37°C to allow the reaction to complete. Absorbance at 450 nm was then measured using a microplate reader.

### 2.4. shRNA-mediating gene silencing

Three shRNAs targeting non-overlapping sequences of human HSP105 were designed and experimentally validated by Genechem, with the sequences provided in **S1 Table**. The shRNAs were subcloned into the GV493 (hU6-MCS-CBh-gcGFP-IRES-puromycin) vector (Genechem) and co-transfected with a lentiviral packaging plasmid mixture into 293T cells. HaCaT cells were seeded into six-well plates (1×10^5 cells per well) and cultured for 12 hours before transduction. HSP105 shRNA lentivirus was added to cultured HaCaT cells under standard infection conditions. Stable cell lines were selected by culturing the cells in complete medium containing 2.0 μg/mL puromycin for three passages. The efficiency of HSP105 silencing in the stable cell lines was evaluated by qPCR and Western blot analysis. Control cells were transfected with lentivirus encoding a scrambled control shRNA ("shC").

### 2.5. HSP105 overexpression

The full-length HSP105 cDNA sequence was synthesized by Genechem (Shanghai, China), with primer sequences provided in **S1 Table**. The cDNA was cloned into the GV492 lentiviral expression vector (Ubi-MCS-3FLAG-CBh-gcGFP-IRES-puromycin, Genechem). The construct was co-transfected with a lentiviral envelope plasmid mixture into 293T cells to produce *HSP105*-expressing lentivirus. Lentiviral transduction of HaCaT cells and the subsequent screening for stable cell lines were performed similarly to the process used for shRNA-mediated gene silencing.

### 2.6. UVB irradiation

UVB irradiation was carried out using a UVB fluorescent lamp (TL 40W/12 RS SLV/25, Philips, Amsterdam, Netherlands), and monitored with a UV340 Research Radiometer (Beijing Shida Photoelectric Technology, Beijing, China). Prior to irradiation, the culture medium was removed, and the cells were washed three times with PBS. Then, a thin layer of Hank’s Balanced Salt Solution was spread over the cells. HaCaT cells were irradiated with a dose of 10–160 mJ/cm^2^, while controls were treated with the balanced salt solution but were not irradiated. After irradiation, the medium was replaced with fresh medium, with or without 50 μM 2-aminoethoxydiphenyl borate (2-APB), and incubation continued for 12 hours.

### 2.7. Protein extraction and Western blot analysis

Total protein was obtained by lysing cells with RIPA lysis buffer (Beyotime, Jiangsu, China) supplemented with a protease inhibitor cocktail to ensure complete protein extraction. Membrane proteins were isolated using the membrane protein extraction kit (Invent Biotechnologies, Beijing, China), following the manufacturer’s protocol. Proteins were separated by SDS-PAGE using 10% or 12.5% gels and then transferred to polyvinylidene difluoride membranes (0.45 μm). After transfer, the membranes were blocked for 15 minutes using a protein-free rapid blocking buffer (P0240, Beyotime, Jiangsu, China). The membranes were then incubated with primary antibodies at 4°C overnight. On the following day, the membranes were washed three times for five minutes each with tris-buffered saline with Tween-20 (TBST). Next, the membranes were incubated with secondary antibodies for one hour, followed by three five-minute washes. Membrane images were acquired using a fusion imaging system. The primary antibodies used in Western blotting included: rabbit anti-HSP105 (1:5000; Abcam), rabbit anti-ORAI1 (1:1000; ProSci), mouse anti-STIM1 (1:500; Santa Cruz Biotechnology), mouse anti-OCLN (1:2000; Proteintech), rabbit anti-CLDN1 (1:2000; Proteintech), rabbit anti-CLDN4 (1:2000; Proteintech), mouse anti-GAPDH (1:5000; Proteintech), rabbit anti-AUBA1B (1:5000; Proteintech), and rabbit anti-ATP1A1 (1:5000; Proteintech).

### 2.8. Confocal microscopy

Cells were seeded on glass slides. Twenty-four hours after UV irradiation, the cells were fixed for 10 minutes with 4% paraformaldehyde and subsequently permeabilized for 20 minutes with Immunostaining Permeabilization Buffer with Saponin (Beyotime, Jiangsu, China). Following permeabilization, the cells were blocked with 1% BSA for 30 minutes, and then incubated with anti-CLDN1 (1: 100, Santa Cruz Biotechnology), anti-CLDN4 (1: 100, Proteintech), and anti-OCLN (1: 100, Proteintech) for 18 h at 4°C, followed by incubation with Alexa Fluor 555 or 647-conjugated secondary antibodies (1:200, Abcam). Fluorescence images were captured using a confocal laser scanning microscope (Leica, Wetzlar, Germany). Fluorescence intensity was quantified using ImageJ, and the MorphoLibJ plug-in was employed to identify cell membranes [27]. The ratio of the fluorescence area at the cell membrane to the total cellular fluorescence area was used as an indicator of tight junction integrity.

### 2.9. Determination of calcium concentrations

HaCaT cells were trypsinized and seeded onto confocal dishes. The cells were washed and incubated at 37°C with Fluo-4 AM (Beyotime, Jiangsu, China) for 30 minutes. Following staining, the dishes were washed and mounted in a microscope chamber filled with calcium-free HEPES buffer. Thapsigargin (Sigma-Aldrich, St. Louis, MO, USA) was added at 1 minutes to induce endoplasmic reticulum calcium release, followed by the addition of 2 mM calcium buffer at 7 minutes to initiate extracellular calcium influx. Intracellular calcium levels were monitored in real-time by measuring the fluorescence intensity of Fluo-4 AM using an inverted fluorescence microscope (Leica, Wetzlar, Germany) equipped with an sCMOS camera. The medium was changed to 2-APB-containing medium 15 min prior to incubation of the Fluo-4 AM as a control for the SOCE-inhibited group.

### 2.10. Quantitative RT-PCR

Total RNA was extracted from cells using the RNA Easy Fast Kit (Tiangen, Beijing, China). RNA concentration and purity were measured with a NanoDrop 2000 spectrophotometer, and cDNA was synthesized with the FastKing cDNA Kit (Tiangen, Beijing, China) following the manufacturer’s instructions. We used 500 ng of RNA to synthesize cDNA in a 20 μL reaction. cDNA templates (0.6 μL) were used for real-time PCR with the SYBR Green FastReal qPCR PreMix (Tiangen, Beijing, China). *GAPDH* served as the reference gene. Each sample was analyzed in triplicate, and gene expression levels were quantified using the comparative Ct method (2^−ΔΔCt^) to determine fold changes. Primer sequences are provided in **Table 1**.

**Table 1:** Primers used for real‐time PCR.

Gene	Forward primer (from 5’ to 3’)	Reverse primer (from 5’ to 3’)
*HSP105*	AGATTGTTGGAGGCGCTAC	CCTCTGGCTACTGCTTCATC
*ORAI1*	GTCAGCACCAGCGGCATCAC	AAGTGGACGGCGAAGACGATAAAG
*STIM1*	GGGTATCTCTGCGGCGAATGC	TGGTGGTGGTGGTGGTGGTAG
*OCLN*	AGCTTCCATTAACTTCGCCTGTG	TCGCCGCCAGTTGTGTAGTC
*CLDN1*	CCGTGCCTTGATGGTGGTTGG	CATCTTCTGCACCTCATCGTCTTCC
*CLDN4*	ATCGGCAGCAACATTGTCACCTC	CAGCAGCGAGTCGTACACCTTG
*GAPDH*	CACCCACTCCTCCACCTTTGAC	GTCCACCACCCTGTTGCTGTAG

### 2.11. Statistical analysis

Statistical analyses were performed using GraphPad Prism 9.0 (GraphPad Software, Inc., United States). Data are presented as mean ± standard deviation (SD). Differences between two groups were analyzed using the Student’s t-test. For comparisons among multiple groups, one-way analysis of variance (ANOVA) was used. *p* < 0.05 was considered statistically significant.

## 3. Results

### 3.1. Effect of UVB radiation on HSP110 family expression

**Fig 1:**
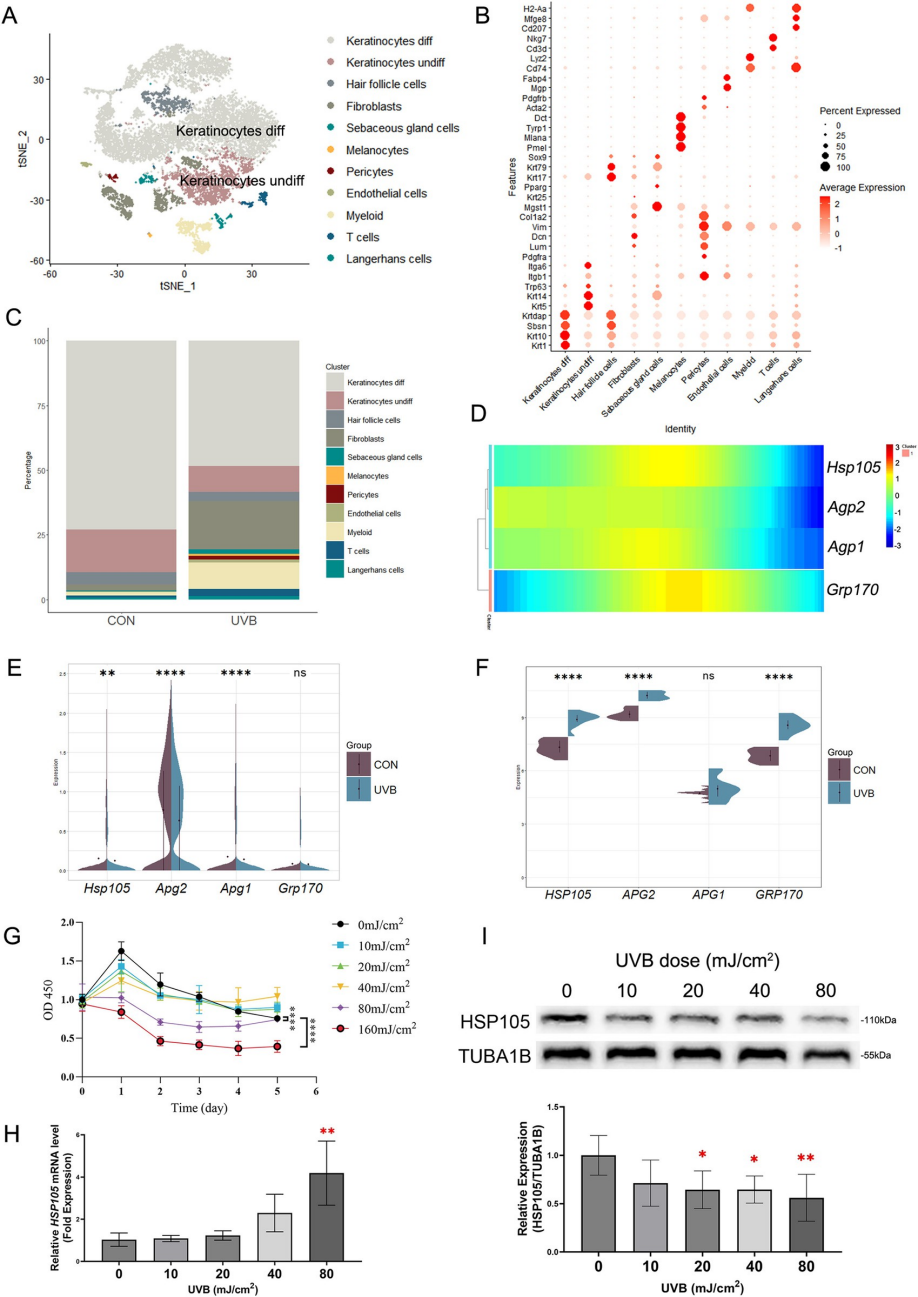
UVB irradiation affects the expression of HSPH family. (A) TSNE plot colored by cells after annotation. (B) Heat map shows the expression of hallmark genes in different cell clusters. The scaled average expression levels of marker genes and the percentage of cells expressing marker genes are expressed by color and size of each dot corresponding to cell clusters, respectively. (C) Stacked bar plot to demonstrate cell percentage between control and UVB samples. (D) Heatmap of HSPH family genes expression along the pseudotime trajectory. (E, F) The violin plots illustrate the expression of HSPH family genes in control and UVB groups from single-cell sequencing data (E) and microarray data (F). (G) The proliferation of HaCaT cells was detected by the CCK-8 method at 0, 24, 48, 72, 96 and 120h after different doses of UVB irradiation. (H) mRNA expression of HSP105 was assessed by quantitative PCR. GAPDH was the internal control. (I) HSP105 was detected by western blot analyses. TUBA1B was the internal control. The number of control samples was adjusted to 1. The experiments were independently repeated at least three times. ** indicates p < 0.005. ns *p* > 0.05, * *p* < 0.05, *** *p* < 0.001, **** *p* < 0.0001.

To characterize HSP110 family gene expression in UVB-induced skin barrier damage, we accessed single-cell RNA sequencing data from the GEO database, including mouse photodamage models and non-irradiated controls. After removing low-quality cells and performing normalization and PCA, we identified 10,317 cells, which were categorized into 22 clusters (**S1 Fig**). By labeling skin cells, we identified 11 cell types, including both undifferentiated and differentiated keratinocytes (**Fig 1A and 1B**). Among these cell types, the proportion of keratinocytes in UVB-irradiated mice was lower than in normal controls (**Fig 1C**), indicating a thinned and damaged skin barrier. As keratinocytes undergo continuous differentiation, we conducted a pseudotemporal analysis of this cell type. **S2 Fig** shows that the nine identified clusters of keratinocytes exhibited a continuous differentiation process. Heatmaps of cell development indicated that *HSP105*, *Apg1* and *Apg2* from the HSP110 family were predominantly expressed during the middle stage of differentiation, while *Grp170* was primarily expressed during the early and middle stages (**Fig 1D**). We used violin plots to visualize changes in HSP110 family gene expression in differentiated keratinocytes following UVB irradiation, revealing that the expression of *HSP105*, *Apg1*, and *Apg2* was significantly reduced in UVB-irradiated keratinocytes compared to the normal group (**Fig 1E**).

Since heat shock protein expression varies significantly between acute and chronic UVB injury, we downloaded the GSE41078 microarray dataset, which focuses on acute UVB-induced injury in human skin. Our analysis demonstrated a significant increase in the expression of HSP105, APG2, and GRP170 following acute UVB irradiation (**Fig 1F**).

To assess the impact of varying acute UVB radiation doses on HaCaT cell viability, we performed in vitro experiments informed by previous studies [8,28]. As illustrated in **Fig 1G**, the CCK-8 assay revealed that low doses of UVB irradiation did not significantly impact HaCaT cell viability. At a dose of 80 mJ/cm^2^, we observed a significant decrease in cell viability, indicating a substantial reduction in proliferative capacity. Additionally, the impact of UVB on HSP105 was confirmed through experimental assays. Compared to the control group, the mRNA levels of *HSP105* increased with higher UVB doses, showing statistically significant differences at 80 mJ/cm^2^ (**Fig 1H**). In contrast, the protein levels of HSP105 were inversely correlated with UVB dose and decreased significantly at doses of 20 mJ/cm^2^ and above (**Fig 1I**).

### 3.2. Effect of UVB on tight junction protein expression and localization

**Fig 2:**
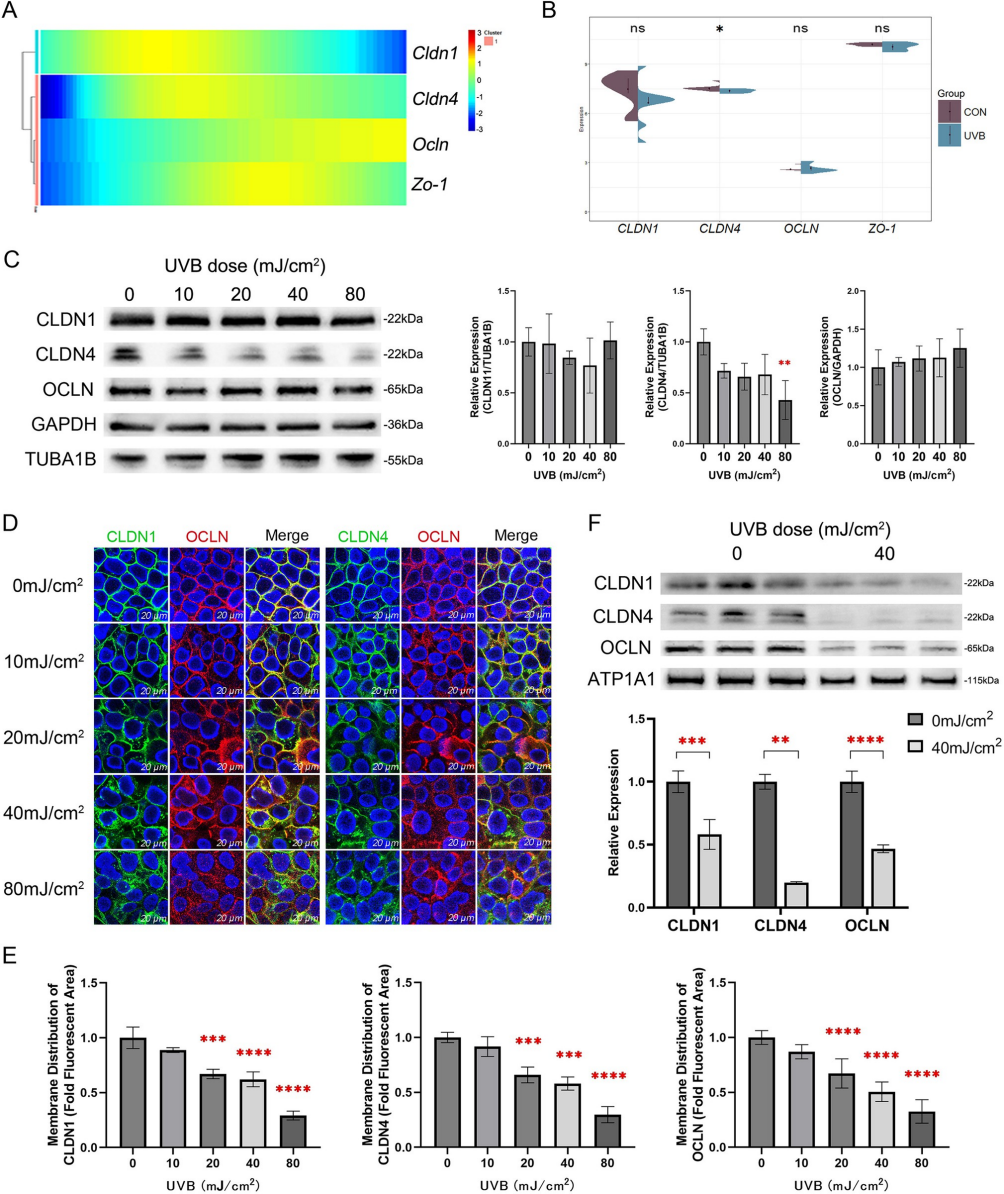
Effect of UVB on the tight junction barrier. (A) Heatmap of Cldn1, Cldn4, Ocln and Zo-1 genes expression along the pseudotime trajectory. (B) The violin plots illustrate the expression levels of CLDN1, CLDN4, OCLN, and ZO-1 in the UVB-exposed and control groups within the acute UVB damage model dataset GSE41078. (C) CLDN1, CLDN4, and OCLN in acute UVB-damaged HaCaT cells were detected by Western blot analysis, with TUBA1B or GAPDH used as internal controls. The experiments were independently repeated three times. (D) Immunofluorescence staining of CLDN1, CLDN4, and OCLN in HaCaT cells 24 hours after exposure to UVB doses ranging from 0 to 80 mJ/cm^2^. Nuclei were stained with DAPI (blue). Scale bar = 20 μm. (E) The fluorescence values of CLDN1, CLDN4 and OCLN at the tight junction are shown as a percentage of the total fluorescence values. The experiments were independently repeated at least three times. (F) Western blot analysis was used to detect CLDN1, CLDN4, and OCLN in the cell membrane, with ATP1A1 serving as an internal control.

First, we analyzed the expression of tight junction proteins Cldn1, Cldn4, Ocln, and Zo-1 in keratinocytes using Single-cell sequencing data. Pseudotime analysis revealed that Cldn1 was predominantly expressed during the early and middle stages of keratinocyte differentiation, whereas Cldn4, Ocln, and Zo-1 were mainly expressed during the middle and late stages (**Fig 2A**). The expression periods of Cldn1, Cldn4, and Ocln were complementary and spanned the entire epidermal layer, which aligns with findings reported by Yuki et al. in human skin [8]. Sequencing data obtained after acute UVB irradiation were analyzed (**Fig 2B**). This analysis revealed that the expression of CLDN1 and CLDN4 was significantly reduced, while the expression of OCLN and ZO-1 remained unchanged.

Subsequently, we measured the protein levels of CLDN1, CLDN4 and OCLN in HaCaT cells subjected to UVB irradiation at doses ranging from 0 to 80 mJ/cm2. The results indicated that only CLDN4 showed a significant decrease following UVB irradiation at the dose of 80 mJ/cm2 (**Fig 2C**).

Damage to tight junctions is primarily characterized by alterations in the distribution of tight junction proteins within the cell membrane [8,29]. We therefore used immunofluorescence to assess changes in the distribution of CLDN1, CLDN4 and OCLN following UVB irradiation at doses from 0 to 80 mJ/cm2. The results revealed that CLDN1, CLDN4 and OCLN exhibited dispersed and fragmented localization in a dose-dependent manner (**Fig 2D and 2E**). Additionally, quantitative analysis of membrane proteins revealed that UVB irradiation at 40 mJ/cm2 resulted in a significant decrease in the distribution of CLDN1, CLDN4 and OCLN within the cell membrane (**Fig 2F**). In summary, we found that acute exposure to UVB reduced mRNA levels of CLDN4, but at the protein level, only a dose of 80 mJ/cm2 resulted in a reduction of CLDN4. In addition, UVB irradiation significantly disrupted the localization of CLDN1, CLDN4, and OCLN in the cell membrane.

### 3.3. Knockdown of HSP105 promotes UVB-induced tight junction injury in HaCaT cells, while ectopic overexpression of HSP105 prevents it

**Fig 3:**
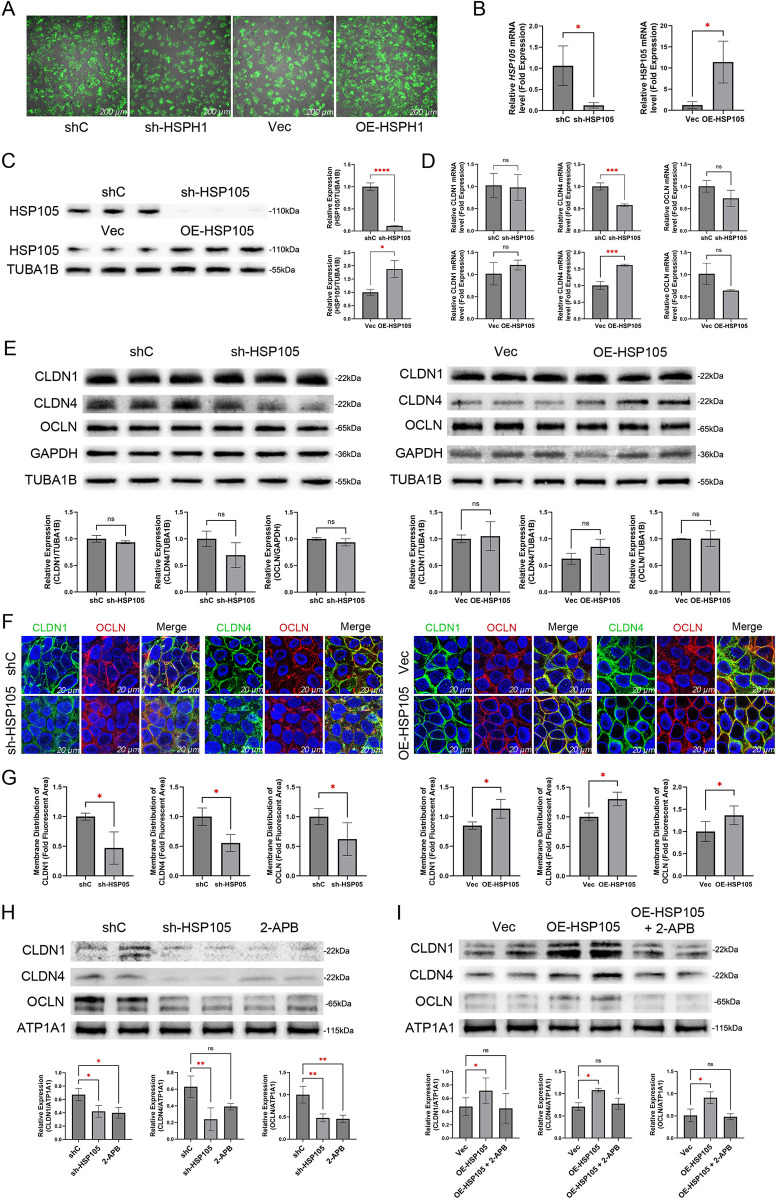
HSP110 expression has an impact on the tight junction barrier. (A) Fluorescence images show the efficiency of lentiviral transfection. Scale bar = 200 μm. (B, C) mRNA expression of HSP105 after shRNA-mediated gene silencing or lentivirus-mediated overexpression (B) and Western blot analysis (C). (D) mRNA expression of CLDN1, CLDN4 and OCLN after HSP105 silencing or overexpression. (E) Western blot analysis of CLDN1, CLDN4, and OCLN following HSP105 silencing (top row) or overexpression (bottom row). (F) Immunofluorescence staining of CLDN1, CLDN4, and OCLN in sh-HSP105, OE-HSP105 and control HaCaT cells 24 hours after exposure to UVB dose of 40mJ/cm^2^. Nuclei were stained with DAPI (blue). Scale bar = 20 μm. (G) The fluorescence values of CLDN1, CLDN4 and OCLN at the tight junction are shown as a percentage of the total fluorescence values. The experiments were independently repeated three times. (H, I) Following 24 hours of UVB irradiation at 40 mJ/cm^2^, Western blot analysis was performed to detect CLDN1, CLDN4, and OCLN in the cell membranes of HSP105-silenced (G) and HSP105-overexpressing cells (H), using ATP1A1 as a internal control.

To investigate the role of HSP105 in UV-induced tight junction damage, we employed shRNA and lentivirus-mediated overexpression approaches. Three lentiviral shRNAs targeting non-overlapping sequences of human HSP105 (105140–1, 105141–1, and 105142–2) were individually transduced into HaCaT cells. Stable cell lines were established by selecting for puromycin resistance. qPCR assays confirmed that HSP105 mRNA was reduced by over 85% in all three HSP105 shRNA-stabilized HaCaT cell lines; thus, we randomly selected the 105140–1 line for further experiments (**Fig 3A and 3B**). Additionally, HSP105 protein levels were significantly downregulated in these cells (**Fig 3C**). We hypothesized that ectopic overexpression of HSP105 (OE-HSP105) would counteract these effects. To test this, we transfected HaCaT cells with lentiviral constructs encoding full-length HSP105 cDNA and established stable OE-HSP105 cells through selection (**Fig 3A**). Both HSP105 mRNA and protein levels were significantly elevated in OE-HSP105 cells compared to control cells containing an empty vector (“Vec”) (**Fig 3B and 3C**).

Subsequently, we detected the mRNA and protein levels of tight junction proteins by qPCR and Western blotting. The results showed that HSP105 shRNA significantly decreased the mRNA level of CLDN4 but had no effect on the protein level. In contrast, ectopic overexpression of HSP105 increased the mRNA level of CLDN4. Neither HSP105 shRNA nor ectopic overexpression affected the mRNA and protein levels of CLDN1 and OCLN (**Fig 3D and 3E**).

Additionally, we employed immunofluorescence and cell membrane protein immunoblotting to examine the distribution of CLDN1, CLDN4, and OCLN in HaCaT cells following UVB irradiation at 40 mJ/cm^2^. The results demonstrated that HSP105 shRNA exacerbated tight junction protein fragmentation after UVB exposure, whereas ectopic overexpression of HSP105 partially mitigated this fragmentation (**Fig 3F**). Similarly, HSP105 shRNA promoted a reduction in tight junction proteins in cell membranes post-UVB irradiation (**Fig 3H**), while ectopic overexpression of HSP105 partially reversed this reduction (**Fig 3I**). In conclusion, the effects of HSP105 on the protein levels of CLDN1, CLDN4 and OCLN were not significant. However, HSP105 partially prevented the damage caused by UVB on the cell membrane localization of CLDN1, CLDN4 and OCLN.

### 3.4. Silencing HSP105 expression abolished store-operated calcium entry

**Fig 4:**
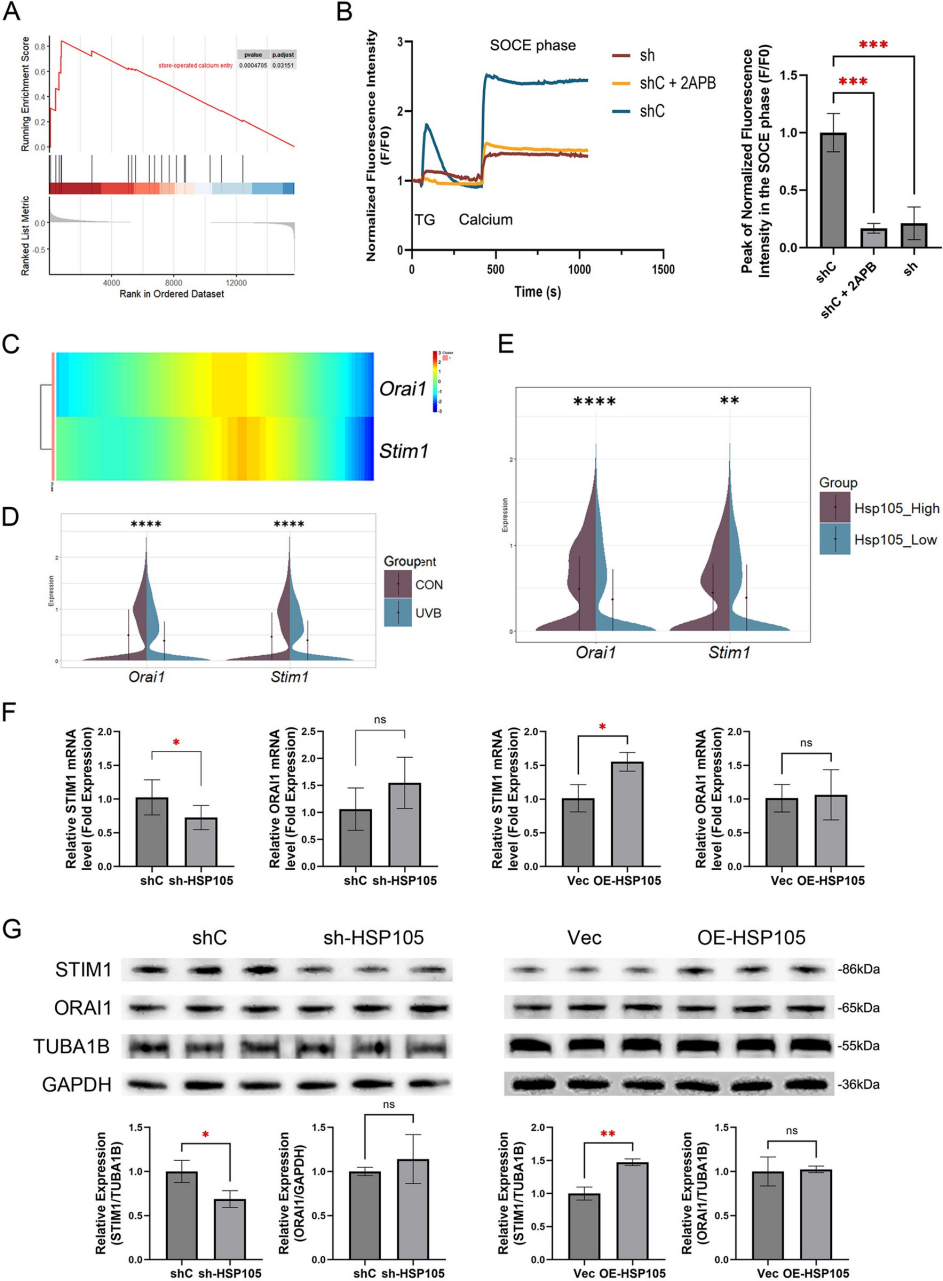
HSP110 mediate TG-induced intracellular calcium mobilization. (A) The results of GSEA demonstrate significant enrichment in Store-operated Calcium Entry. (B) Cytosolic Ca2+ levels in sh-HSP105 HaCaT cells. Extracellular Ca2+ was removed, followed by the addition of thapsigargin (TG) (2μM) for Ca2+ depletion in the ER. Ca2+ (1.8 mM) was then added to the extracellular fluid, and SOCE-induced Ca2+ elevation was observed. The group treated with 2-APB served as a positive control for SOCE inhibition. Bar plot shows the peak fluorescence intensity in the SOCE phase. The experiments were independently repeated three times. (C) Heatmap of Orai1 and Stim1 genes expression along the pseudotime trajectory. (D) The violin plots illustrate the expression of Orai1 and Stim1 in control and UVB groups from single-cell sequencing data. (E) Violin plots based on single-cell sequencing data display the expression levels of Orai1 and Stim1 in HSP105 low-expression and high-expression groups. Groups were stratified based on the median value of HSP105. (F) mRNA expression of ORAI1 and STIM1 were assessed by quantitative PCR. (G) ORAI1 and STIM1 were detected by western blot analyses. TUBA1B or GAPDH were the internal control.

To investigate the intrinsic mechanism of HSP105 in maintaining tight junction localization, we used scRNA sequencing data to analyze potential downstream mechanisms. Through GSEA, we identified 10 Gene Ontology terms closely related to HSP105, including SOCE (**Figs 4A and S3**). Since calcium concentration gradient is required for epidermal differentiation, and calcium signaling is crucial for regulating cell survival and gene expression [19], we hypothesized that HSP105 regulates SOCE, thus influencing the integrity of tight junctions. In the experiments, endoplasmic reticulum calcium depletion was induced by treating cells with 2 μM Thapsigargin (TG) in a calcium-free solution. Calcium influx was detected following the addition of 2 mM calcium solution. As shown in **Fig 4B**, calcium influx was attenuated in sh-HSP105 cells. Stim1 and Orai1 are key regulators of SOCE, so we analyzed their expression in Keratinocytes using scRNA sequencing data. Pseudotime analysis revealed that Stim1 and Orai1 were primarily expressed during the early and middle stages of keratinocyte differentiation, aligning with the expression timeline of Hsp105 (**Fig 4C**). After UVB irradiation, the expression of both Stim1 and Orai1 significantly decreased in mouse keratinocytes (**Fig 4D**). Keratinocytes were divided into high and low expression groups based on the median value of Hsp105 expression, with a significant decrease in both Stim1 and Orai1 observed in the low expression group (**Fig 4E**). In in vitro experiments, the levels of STIM1 and ORAI1 were further evaluated by qPCR and western blotting in sh-HSP105, OE-HSP105 and control HaCaT cells. Transcript levels of STIM1 were reduced in sh-HSP105 cells and elevated in OE-HSP105 cells. However, there was no significant change in the transcript levels of ORAI1 (**Fig 4F**). Changes in the protein levels of STIM1 and ORAI1 mirrored the observed changes in their transcript levels (**Fig 4G**). In summary, these results suggest that HSP105 levels may influence both the transcript and protein levels of STIM1, but do not significantly affect ORAI1.

### 3.5. HSP105 reduces UVB-induced tight junction damage by protecting the SOCE

**Fig 5:**
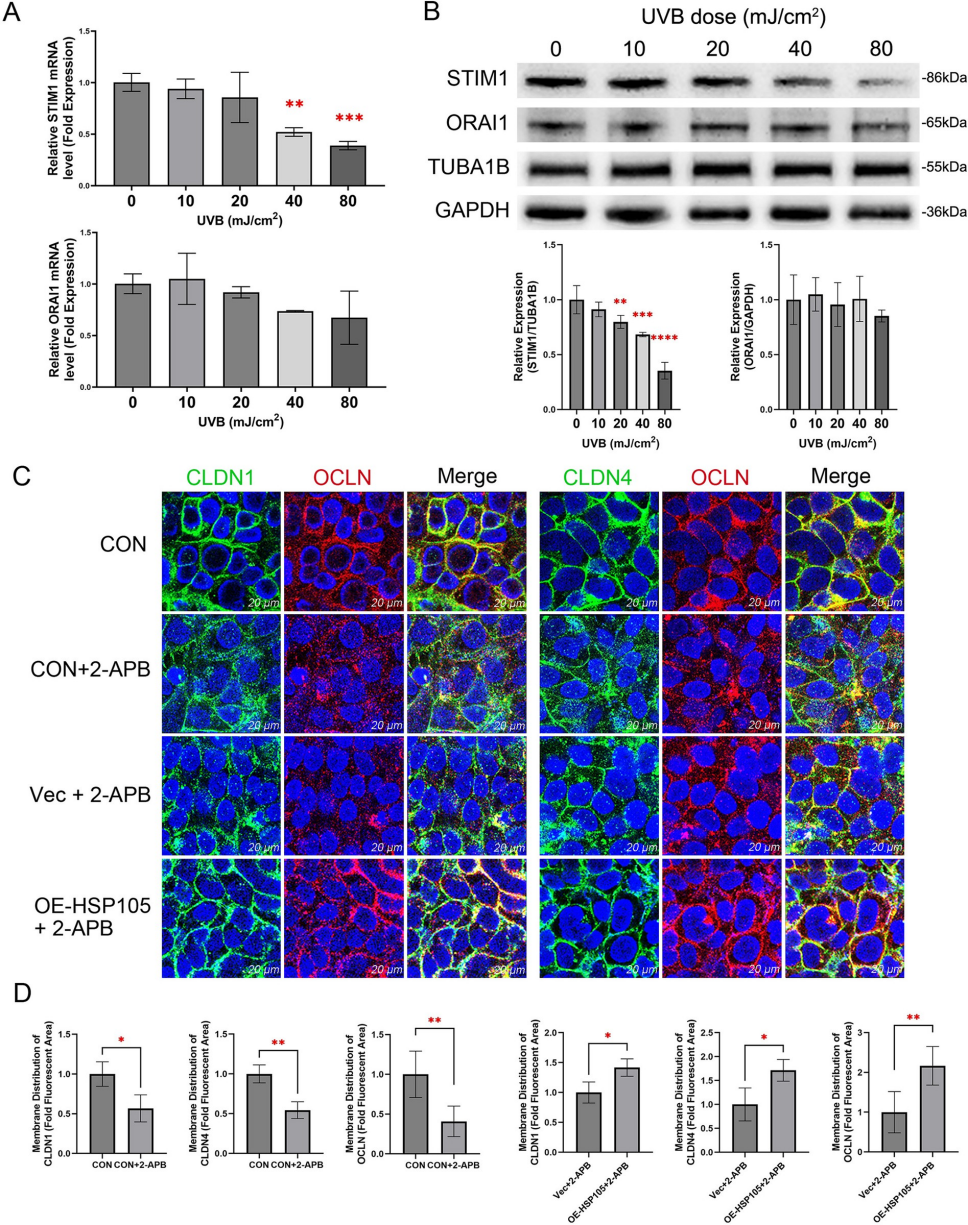
The role of SOCE in mediating UVB-induced tight junction barrier damage. (A) mRNA expression of ORAI1 and STIM1 were assessed by quantitative PCR. (B) ORAI1 and STIM1 were detected by western blot analyses. TUBA1B or GAPDH were the internal control. (C) Immunofluorescence staining of CLDN1, CLDN4, and OCLN in HaCaT cells 24 hours after exposure to 40 mJ/cm^2^ UVB, following SOCE inhibition with 2-APB and lentiviral overexpression. Nuclei are stained with DAPI (blue). Scale bar = 20 μm. (D) The fluorescence values of CLDN1, CLDN4 and OCLN at the tight junction are shown as a percentage of the total fluorescence values. The experiments were independently repeated three times.

To further investigate the role of SOCE in the protective effect of HSP105 against UVB-induced tight junction damage, we first assessed the transcriptional and protein levels of STIM1 and ORAI1 following 0–80 mJ/cm^2^ UVB irradiation in vitro. The results showed that the mRNA level of STIM1 decreased significantly at UVB doses of 40 mJ/cm^2^ and 80 mJ/cm^2^. However, the mRNA level of ORAI1 did not show a significant reduction (**Fig 5A**). Consistent with the transcript levels, the protein levels of STIM1 also decreased with increasing UVB dose, and were statistically different at UVB doses of 20 mJ/cm^2^ and above (**Fig 5B**).

2-APB is a well-known SOCE inhibitor that directly inhibits ORAI1 channels at a concentration of 50 μM. Additionally, it suppresses STIM1 protein by enhancing intramolecular interactions between the CC1 and SOAR regions of STIM1 [30]. Therefore, we added 50 μM 2-APB to the culture medium 15 minutes before UVB irradiation to explore the effect of SOCE inhibition on tight junction damage. As shown in **Fig 5C**, after 40 mJ/cm^2^ UVB irradiation, the tight junctions in the 2-APB group appeared more dispersed and fragmented compared to those in the group exposed to UVB alone. Additionally, the HSP105 overexpression significantly alleviated the dispersion and fragmentation of tight junctions induced by 2-APB.

Additionally, Western bloting quantification experiments were performed using extracted membrane proteins. The results showed that adding 2-APB to the culture medium before UVB irradiation at 40 mJ/cm^2^ led to a significant decrease in the distribution of CLDN1, CLDN4, and OCLN in the cell membranes of shC group cells, similar to what was observed in the sh-HSP05 group (**Fig 3G**). Moreover, incubation of OE-HSP105 group cells with 2-APB prevented the increased distribution of CLDN1, CLDN4, and OCLN in the cell membrane (**Fig 3H**). These results suggest that HSP105 may exert its effect on tight junctions by protecting the function of SOCE, and that STIM1 is a key factor mediating the protective effect of HSP105 on SOCE.

## 4. Discussion

Tight junction damage is common in skin diseases. For instance, a premature stop codon in CLDN1 has been identified in neonatal ichthyosis [31]. As a key component of the skin barrier, tight junction damage may be a precursor to immune and allergic skin disorders [4]. Among the significant environmental factors affecting the epidermis, UVB radiation from sunlight plays a prominent role [32].

In our study, we initially investigated the effects of acute UVB exposure on the expression and distribution of tight junction proteins. The results showed that acute UV exposure primarily disrupted the membrane distribution of tight junction proteins. After UVB exposure, CLDN4 transcript levels decreased. However, following acute exposure to low-dose UVB (which did not significantly affect cell viability), no reduction in the protein levels of CLDN1, CLDN4, or OCLN was observed. This finding is consistent with the results of Yuki et al [8]. The reason for the discrepancy between the transcription and protein levels of tight junction proteins remains unclear. We propose that CLDN4 is more susceptible to UVB-induced damage compared to CLDN1 and OCLN, with this susceptibility initially reflected in reduced transcript levels.

Next, we examined the changes in HSP105 expression following both acute and chronic UVB exposure. In our study, sequencing data obtained 24 hours after acute UV irradiation revealed elevated transcript levels of HSP105, Apg2, and GRP170. In vitro experiments further demonstrated that the increase in HSP105 transcript levels was dose-related. Conversely, sequencing data from chronic UVB exposure demonstrated a significant decrease in transcript levels of HSP105, Apg2, and Apg1 in the HSPH family. The expression levels of UVB-induced heat shock proteins are dynamic, exhibiting significant variation between chronic and acute UVB exposure [10,33,34]. The rapid increase in expression during the acute phase may reflect an immediate response to harmful stimuli, while later phases are likely associated with adaptation or recovery from stress [35].

The protein levels of HSP105 decreased in a dose-dependent manner following acute UVB exposure, contrasting with the increase in its transcript levels. This discrepancy highlights the limitations of using transcription levels as a proxy for protein abundance, particularly under stress conditions [36,37]. Although our study does not fully elucidate the reasons behind the discrepancy between HSP105 protein levels and mRNA expression, it provides evidence suggesting that UVB leads to reduced HSP105 protein levels through a non-transcriptional pathway.

The role of the HSPH family members, especially HSP105, in regulating the tight junctions remains inadequately understood. In MDCK renal epithelial cells, Apg2, a chaperone for ZO-1, was observed to co-localize at cell junctions and assist in the assembly of ZO-1 [16,38]. Specific knockout of Apg1 in the cortical segment of distal tubular epithelial cells may disrupt the renal epithelial barrier, making mice more susceptible to osmotic stress [39].

Our study demonstrated that UVB-induced disruption of CLDN1, CLDN4, and OCLN distribution in keratinocytes was exacerbated by silencing HSP105, while overexpressing HSP105 mitigated this impairment. Interestingly, silencing HSP105 only reduced the transcript levels of CLDN4, with no effect on CLDN1 or OCLN. This suggests that HSP105 may be involved in the regulation of the transcriptional level of CLDN4, but has no effect on CLDN1 and OCLN.

At the protein level, neither silencing nor overexpressing HSP105 affected CLDN1, CLDN4, or OCLN. These results suggest that HSP105 does not primarily protect against UVB-induced tight junction damage by regulating transcript levels or preventing protein degradation. Instead, HSP105’s protective role in tight junctions seems to be mainly related to maintaining their functional distribution. To further investigate the potential downstream pathways through which HSP105 protects tight junctions in the UVB damage model, we performed GSEA enrichment analysis. Notably, the SOCE pathway was significantly enriched.

Given that calcium ions are involved in the localization and formation of tight junctions, and SOCE is crucial for maintaining intracellular calcium levels, we conducted further investigations focusing on the SOCE pathway [40]. Our findings suggested that UV exposure may impair SOCE by affecting the transcription levels of STIM1, while ORAI1 remains unaffected. We also found that silencing HSP105 significantly inhibited Ca2+ influx following ER Ca2+ depletion, likely through the downregulation of STIM1 expression. Additionally, the SOCE inhibitor 2-APB exacerbated the disruption of CLDN1, CLDN4, and OCLN distribution after UVB exposure, whereas HSP105 overexpression partially alleviated this effect, indicating that SOCE plays a role in HSP105-mediated protection against UVB-induced tight junction damage. Based on these results, we propose that in acute UVB damage, HSP105 functions as a regulatory protein, potentially acting as a transcription factor or kinase to modulate stress response pathways or expression cascades [35]. The reduction of HSP105 leads to STIM1 transcriptional downregulation, weakening SOCE and ultimately disrupting intracellular calcium homeostasis, resulting in impaired functional distribution of tight junctions. However, HSP105 does not appear to regulate the transcript levels of ORAI1.

Although we combined online dataset analysis with experimental data, our findings are still largely based on in vitro studies. Therefore, further validation of HSP105’s protective role against UVB-induced tight junction damage through in vivo experiments will be necessary. To investigate SOCE’s role in HSP105-mediated protection against tight junction injury, we employed the commonly used SOCE inhibitor, 2-APB. However, we cannot rule out the possibility that 2-APB inhibits other pathways, including IP3-induced Ca2+ release, transient receptor potential cation channels, potassium channels, and volume-regulated anion channels, which may also affect tight junction integrity [41–44]. Developing and using more specific SOCE inhibitors would aid future research. Additionally, further studies are required to investigate other mechanisms through which HSP105 protects the skin barrier, beyond SOCE.

In conclusion, the present study demonstrated that acute UVB exposure results in impaired functional distribution of tight junction proteins, including CLDN1, CLDN4, and OCLN, with variable expressions of transcription and protein levels. Furthermore, acute UVB exposure was shown to reduce HSP105 protein levels, resulting in decreased STIM1 expression and impaired SOCE, which subsequently contributes to damage to the functional distribution of tight junction proteins. Conversely, the overexpression of HSP105 was demonstrated to mitigate the deleterious effects observed. These findings highlight the regulatory function of HSP105 in tight junctions and suggest potential therapeutic strategies for addressing damage to the skin barrier.

## Supporting information

S1 Fig The 22 cell clusters categorized using t-SNE. (PDF)

S2 Fig Differentiation trajectory results for keratinocytes. (PDF)

S3 Fig Top 10 GSEA results. (PDF)

S1 Table shRNA and HSPH1 overexpression sequences utilized in this study. (PDF)

## References

[1] Yoshida K, Yokouchi M, Nagao K, Ishii K, Amagai M, Kubo A. Functional tight junction barrier localizes in the second layer of the stratum granulosum of human epidermis. J Dermatol Sci. 2013 Aug;71(2):89–99. doi: 10.1016/j.jdermsci.2013.04.021 23712060

[2] Riethmüller C. Assessing the skin barrier via corneocyte morphometry. Exp Dermatol. 2018 Aug;27(8):923–30. doi: 10.1111/exd.13741 30019542

[3] Kiatsurayanon C, Ogawa H, Niyonsaba F. The Role of Host Defense Peptide Human β-defensins in the Maintenance of Skin Barriers. Curr Pharm Des. 2018;24(10):1092–9. 29589537 10.2174/1381612824666180327164445

[4] Egawa G, Kabashima K. Multifactorial skin barrier deficiency and atopic dermatitis: Essential topics to prevent the atopic march. J Allergy Clin Immunol. 2016 Aug;138(2):350–358.e1. doi: 10.1016/j.jaci.2016.06.002 27497277

[5] Sugawara T, Iwamoto N, Akashi M, Kojima T, Hisatsune J, Sugai M, et al. Tight junction dysfunction in the stratum granulosum leads to aberrant stratum corneum barrier function in claudin-1-deficient mice. J Dermatol Sci. 2013 Apr;70(1):12–8. doi: 10.1016/j.jdermsci.2013.01.002 23433550

[6] Saito AC, Higashi T, Fukazawa Y, Otani T, Tauchi M, Higashi AY, et al. Occludin and tricellulin facilitate formation of anastomosing tight-junction strand network to improve barrier function. Mol Biol Cell. 2021 Apr 15;32(8):722–38. doi: 10.1091/mbc.E20-07-0464 33566640 PMC8108510

[7] Bäsler K, Bergmann S, Heisig M, Naegel A, Zorn-Kruppa M, Brandner JM. The role of tight junctions in skin barrier function and dermal absorption. J Control Release. 2016 Nov 28;242:105–18. doi: 10.1016/j.jconrel.2016.08.007 27521894

[8] Yuki T, Hachiya A, Kusaka A, Sriwiriyanont P, Visscher MO, Morita K, et al. Characterization of tight junctions and their disruption by UVB in human epidermis and cultured keratinocytes. J Invest Dermatol. 2011 Mar;131(3):744–52. doi: 10.1038/jid.2010.385 21160495

[9] Richa null Sinha RP, Häder DP. Physiological aspects of UV-excitation of DNA. Top Curr Chem. 2015;356:203–48. 24696352 10.1007/128_2014_531

[10] Wang ZY, Li A, Huang X, Bai GL, Jiang YX, Li RL, et al. HSP27 Protects Skin From Ultraviolet B -Induced Photodamage by Regulating Autophagy and Reactive Oxygen Species Production. Front Cell Dev Biol. 2022;10:852244. doi: 10.3389/fcell.2022.852244 35445017 PMC9014213

[11] Wang ZY, Li A, Huang X, Bai GL, Jiang YX, Li RL, et al. HSP27 Protects Skin From Ultraviolet B -Induced Photodamage by Regulating Autophagy and Reactive Oxygen Species Production. Front Cell Dev Biol. 2022;10:852244. doi: 10.3389/fcell.2022.852244 35445017 PMC9014213

[12] Vos MJ, Hageman J, Carra S, Kampinga HH. Structural and functional diversities between members of the human HSPB, HSPH, HSPA, and DNAJ chaperone families. Biochemistry. 2008 Jul 8;47(27):7001–11. doi: 10.1021/bi800639z 18557634

[13] Muchemwa FC, Nakatsura T, Ihn H, Kageshita T. Heat shock protein 105 is overexpressed in squamous cell carcinoma and extramammary Paget disease but not in basal cell carcinoma: HSP105 is overexpressed in skin cancers. British Journal of Dermatology. 2006 Sep;155(3):582–5. 16911285 10.1111/j.1365-2133.2006.07362.x

[14] Maytin EV. Differential effects of heat shock and UVB light upon stress protein expression in epidermal keratinocytes. J Biol Chem. 1992 Nov 15;267(32):23189–96. 1429666

[15] Rampelt H, Kirstein-Miles J, Nillegoda NB, Chi K, Scholz SR, Morimoto RI, et al. Metazoan Hsp70 machines use Hsp110 to power protein disaggregation. EMBO J. 2012 Nov 5;31(21):4221–35. doi: 10.1038/emboj.2012.264 22990239 PMC3492728

[16] Tsapara A, Matter K, Balda MS. The heat-shock protein Apg-2 binds to the tight junction protein ZO-1 and regulates transcriptional activity of ZONAB. Mol Biol Cell. 2006 Mar;17(3):1322–30. doi: 10.1091/mbc.e05-06-0507 16407410 PMC1382320

[17] Putney JW. A model for receptor-regulated calcium entry. Cell Calcium. 1986 Feb;7(1):1–12. doi: 10.1016/0143-4160(86)90026-6 2420465

[18] Berridge MJ, Bootman MD, Lipp P. Calcium—a life and death signal. Nature. 1998 Oct 15;395(6703):645–8. doi: 10.1038/27094 9790183

[19] Bikle DD, Xie Z, Tu CL. Calcium regulation of keratinocyte differentiation. Expert Rev Endocrinol Metab. 2012 Jul;7(4):461–72. doi: 10.1586/eem.12.34 23144648 PMC3491811

[20] Magee AI, Lytton NA, Watt FM. Calcium-induced changes in cytoskeleton and motility of cultured human keratinocytes. Exp Cell Res. 1987 Sep;172(1):43–53. doi: 10.1016/0014-4827(87)90091-7 2443374

[21] Liu T, Juan Z, Xia B, Ren G, Xi Z, Hao J, et al. HSP70 protects H9C2 cells from hypoxia and reoxygenation injury through STIM1/IP3R. Cell Stress Chaperones. 2022 Sep;27(5):535–44. doi: 10.1007/s12192-022-01290-0 35841499 PMC9485396

[22] Huang CY, Wei PL, Chen WY, Chang WC, Chang YJ. Silencing Heat Shock Protein 27 Inhibits the Progression and Metastasis of Colorectal Cancer (CRC) by Maintaining the Stability of Stromal Interaction Molecule 1 (STIM1) Proteins. Cells. 2018 Dec 10;7(12):262. doi: 10.3390/cells7120262 30544747 PMC6315635

[23] Crispin MK, Fuentes-Duculan J, Gulati N, Johnson-Huang LM, Lentini T, Sullivan-Whalen M, et al. Gene profiling of narrow-band UVB-induced skin injury defines cellular and molecular innate immune responses. J Invest Dermatol. 2013 Mar;133(3):692–701. doi: 10.1038/jid.2012.359 23151847 PMC3679916

[24] Lin Y, Cao Z, Lyu T, Kong T, Zhang Q, Wu K, et al. Single-cell RNA-seq of UVB-radiated skin reveals landscape of photoaging-related inflammation and protection by vitamin D. Gene. 2022 Jul 15;831:146563. doi: 10.1016/j.gene.2022.146563 35577040

[25] Ma F, Plazyo O, Billi AC, Tsoi LC, Xing X, Wasikowski R, et al. Single cell and spatial sequencing define processes by which keratinocytes and fibroblasts amplify inflammatory responses in psoriasis. Nat Commun. 2023 Jun 12;14(1):3455. doi: 10.1038/s41467-023-39020-4 37308489 PMC10261041

[26] Liberzon A, Birger C, Thorvaldsdóttir H, Ghandi M, Mesirov JP, Tamayo P. The Molecular Signatures Database (MSigDB) hallmark gene set collection. Cell Syst. 2015 Dec 23;1(6):417–25. doi: 10.1016/j.cels.2015.12.004 26771021 PMC4707969

[27] Legland D, Arganda-Carreras I, Andrey P. MorphoLibJ: integrated library and plugins for mathematical morphology with ImageJ. Bioinformatics. 2016 Nov 15;32(22):3532–4. doi: 10.1093/bioinformatics/btw413 27412086

[28] Kim DJ, Iwasaki A, Chien AL, Kang S. UVB-mediated DNA damage induces matrix metalloproteinases to promote photoaging in an AhR- and SP1-dependent manner. JCI Insight. 7(9):e156344. doi: 10.1172/jci.insight.156344 35316219 PMC9090247

[29] Jiang Y, Song J, Xu Y, Liu C, Qian W, Bai T, et al. Piezo1 regulates intestinal epithelial function by affecting the tight junction protein claudin-1 via the ROCK pathway. Life Sciences. 2021 Jun 15;275:119254. doi: 10.1016/j.lfs.2021.119254 33636174

[30] Wei M, Zhou Y, Sun A, Ma G, He L, Zhou L, et al. Molecular mechanisms of inhibition on STIM1-Orai1 mediated Ca2+ entry induced by 2-aminoethoxydiphenyl borate. Pflugers Arch. 2016 Nov;468(11–12):2061–74. 27726010 10.1007/s00424-016-1880-z PMC5393042

[31] Hadj-Rabia S, Baala L, Vabres P, Hamel-Teillac D, Jacquemin E, Fabre M, et al. Claudin-1 gene mutations in neonatal sclerosing cholangitis associated with ichthyosis: a tight junction disease. Gastroenterology. 2004 Nov;127(5):1386–90. doi: 10.1053/j.gastro.2004.07.022 15521008

[32] Alhasaniah A, Sherratt MJ, O’Neill CA. The Impact of Ultraviolet Radiation on Barrier Function in Human Skin: Molecular Mechanisms and Topical Therapeutics. Curr Med Chem. 2018;25(40):5503–11. doi: 10.2174/0929867324666171106164916 29110595

[33] Liu Y, Huang X, Wang P, Pan Y, Cao D, Liu C, et al. The effects of HSP27 against UVB-induced photoaging in rat skin. Biochem Biophys Res Commun. 2019 May 7;512(3):435–40. doi: 10.1016/j.bbrc.2019.03.076 30902393

[34] Shirato K, Takanari J, Koda T, Sakurai T, Ogasawara J, Ohno H, et al. A standardized extract of Asparagus officinalis stem prevents reduction in heat shock protein 70 expression in ultraviolet-B-irradiated normal human dermal fibroblasts: an in vitro study. Environ Health Prev Med. 2018 Aug 21;23(1):40. doi: 10.1186/s12199-018-0730-3 30131067 PMC6104003

[35] Richter K, Haslbeck M, Buchner J. The Heat Shock Response: Life on the Verge of Death. Molecular Cell. 2010 Oct 22;40(2):253–66. doi: 10.1016/j.molcel.2010.10.006 20965420

[36] Wu B, Qiao J, Wang X, Liu M, Xu S, Sun D. Factors affecting the rapid changes of protein under short-term heat stress. BMC Genomics. 2021 Apr 13;22(1):263. doi: 10.1186/s12864-021-07560-y 33849452 PMC8042900

[37] Lewis M, Götting M, Anttila K, Kanerva M, Prokkola JM, Seppänen E, et al. Different Relationship between hsp70 mRNA and hsp70 Levels in the Heat Shock Response of Two Salmonids with Dissimilar Temperature Preference. Front Physiol. 2016;7:511. doi: 10.3389/fphys.2016.00511 27872596 PMC5098114

[38] Aijaz S, Sanchez-Heras E, Balda MS, Matter K. Regulation of tight junction assembly and epithelial morphogenesis by the heat shock protein Apg-2. BMC Cell Biol. 2007 Nov 20;8:49. doi: 10.1186/1471-2121-8-49 18028534 PMC2211299

[39] Held T, Paprotta I, Khulan J, Hemmerlein B, Binder L, Wolf S, et al. Hspa4l-deficient mice display increased incidence of male infertility and hydronephrosis development. Mol Cell Biol. 2006 Nov;26(21):8099–108. doi: 10.1128/MCB.01332-06 16923965 PMC1636758

[40] Seo SH, Kim SE, Lee SE. ER stress induced by ER calcium depletion and UVB irradiation regulates tight junction barrier integrity in human keratinocytes. Journal of Dermatological Science. 2020 Apr 1;98(1):41–9. doi: 10.1016/j.jdermsci.2020.02.006 32376153

[41] Maruyama T, Kanaji T, Nakade S, Kanno T, Mikoshiba K. 2APB, 2-Aminoethoxydiphenyl Borate, a Membrane-Penetrable Modulator of Ins(1,4,5)P3-Induced Ca2+ Release. The Journal of Biochemistry. 1997 Sep 1;122(3):498–505. doi: 10.1093/oxfordjournals.jbchem.a021780 9348075

[42] Ma KT, Guan BC, Yang YQ, Nuttall AL, Jiang ZG. 2-Aminoethoxydiphenyl borate blocks electrical coupling and inhibits voltage-gated K+ channels in guinea pig arteriole cells. Am J Physiol Heart Circ Physiol. 2011 Jan;300(1):H335–346. doi: 10.1152/ajpheart.00737.2010 21037232 PMC3023242

[43] Chokshi R, Fruasaha P, Kozak JA. 2-aminoethyl diphenyl borinate (2-APB) inhibits TRPM7 channels through an intracellular acidification mechanism. Channels (Austin). 2012;6(5):362–9. doi: 10.4161/chan.21628 22922232 PMC3508775

[44] Lemonnier L, Prevarskaya N, Mazurier J, Shuba Y, Skryma R. 2-APB inhibits volume-regulated anion channels independently from intracellular calcium signaling modulation. FEBS Lett. 2004 Jan 2;556(1–3):121–6. doi: 10.1016/s0014-5793(03)01387-5 14706838

