# Supplementary figures and images for: HSP105 inhibition downregulates store-operated calcium entry and promotes acute UVB-induced tight junction disruption
Source: PLoS One. 2024 Dec 5;19(12):e0314816. doi: 10.1371/journal.pone.0314816 (PMC11620698; doi:10.1371/journal.pone.0314816)

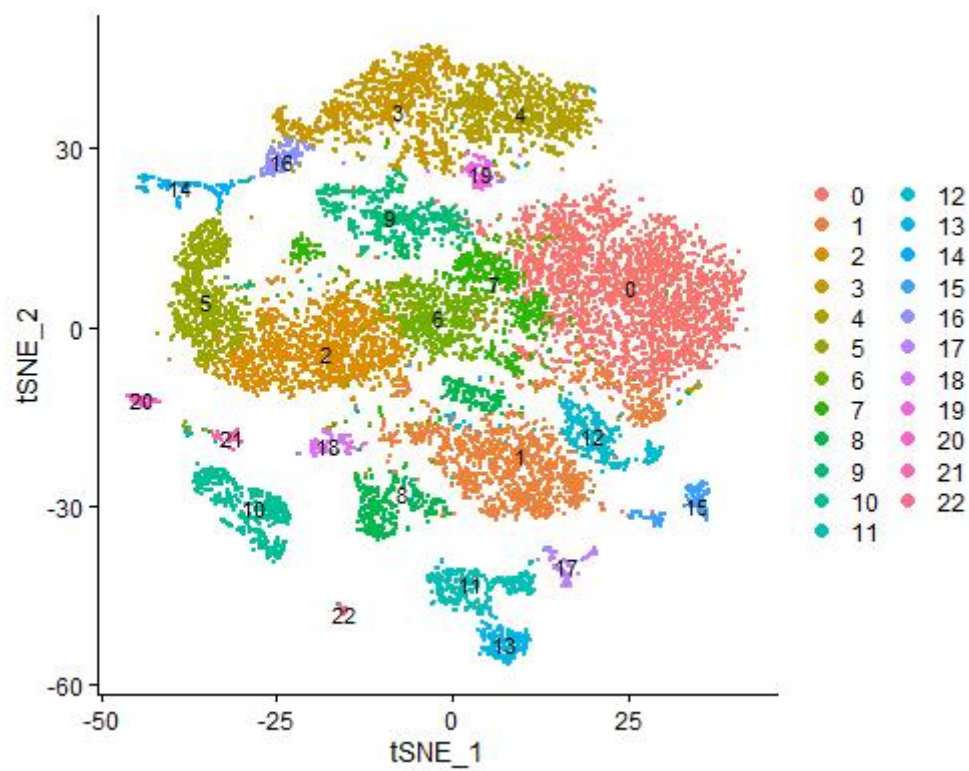

**Figure S1. The 22 cell clusters categorized using t-SNE.**

Supplement: S1 Fig — (PDF) [file pone.0314816.s001.pdf]

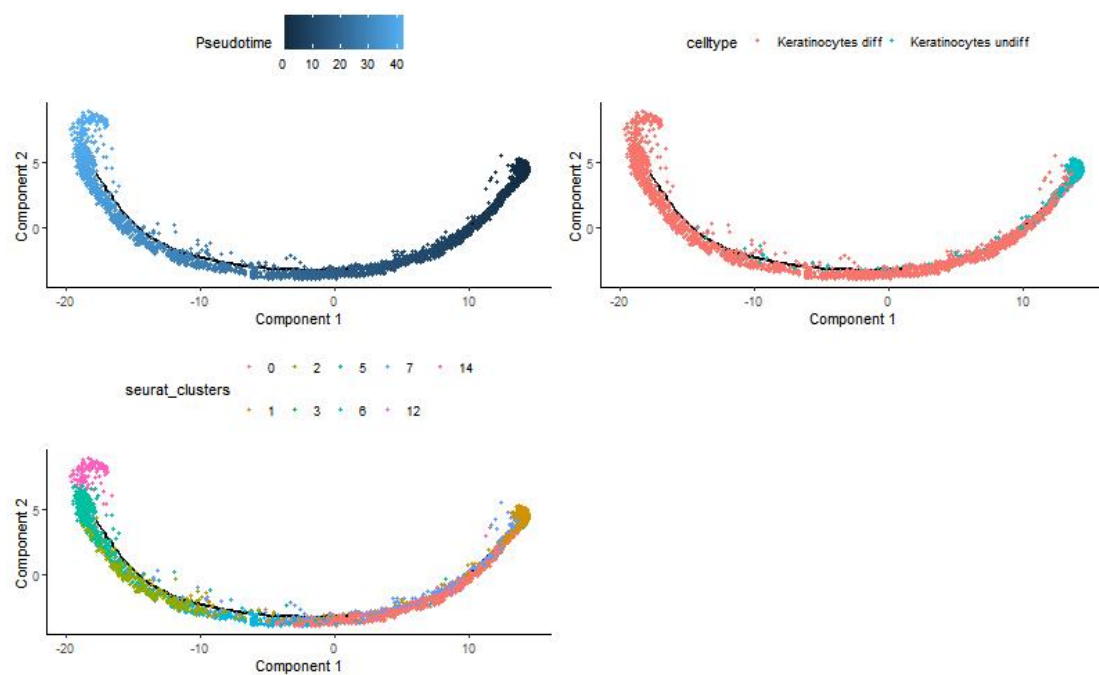

**Figure S2. Differentiation trajectory results for keratinocytes.**

Supplement: S2 Fig — (PDF) [file pone.0314816.s002.pdf]

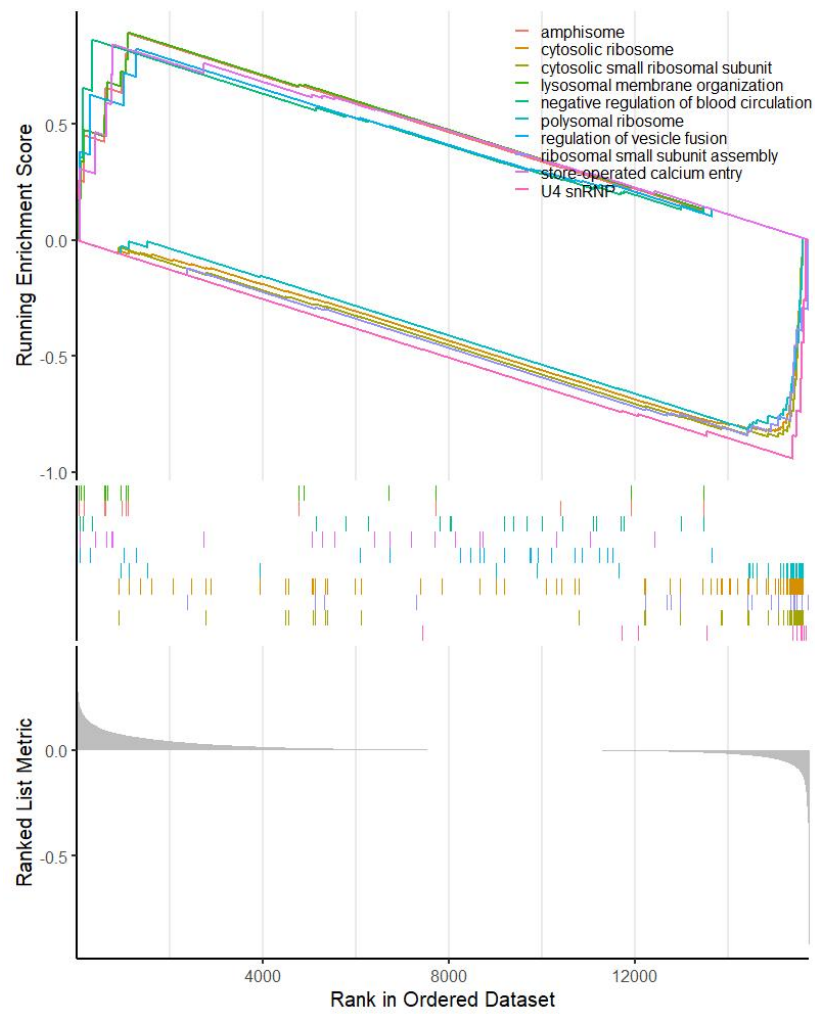

**Figure S3. Top 10 GSEA results.**

Supplement: S3 Fig — (PDF) [file pone.0314816.s003.pdf]
