# Supplementary material for: HSP105 inhibition downregulates store-operated calcium entry and promotes acute UVB-induced tight junction disruption
Source: PLoS One. 2024 Dec 5;19(12):e0314816. doi: 10.1371/journal.pone.0314816 (PMC11620698; doi:10.1371/journal.pone.0314816)
Supplement: S1 Table — (PDF) [file pone.0314816.s004.pdf]

Table S1. shRNA and HSPH1 overexpression sequences utilized in this study

**shRNA**					
NO.	5’	STEM	Loop	STEM	3’
HSPH1-RNAi(105140-1)-a	Ccgg	ccACTGAATATCGAATGCTTT	CTCGA	AAAGC	TTTTTg
HSPH1-RNAi(105140-1)-b	aattcaaaaa	ccACTGAATATCGAATGCTTT	CTCGA	AAAGC	
HSPH1-RNAi(105141-1)-a	Ccgg	ccAGTAACAGATTGTGTTATT	CTCGA	AATAA	TTTTTg
HSPH1-RNAi(105141-1)-b	aattcaaaaa	ccAGTAACAGATTGTGTTATT	CTCGA	AATAA	
HSPH1-RNAi(105142-2)-a	Ccgg	atGAGAAATACAACCATATTG	CTCGA	CAATA	TTTTTg
HSPH1-RNAi(105142-2)-b	aattcaaaaa	atGAGAAATACAACCATATTG	CTCGA	CAATA	

**overexpression**	
ID	seq
HSPH1(73184-3)-p1	AGGTCGACTCTAGAGGATCCCGCCACCATGGCAACGGCGGCCGTTCTCCGGGG
HSPH1(73184-3)-p2	TCCTTGTAGTCCATACCGTCCAAGTCCATATTAACAGAATTTTTC
